# Understanding the influence of left ventricular assist device inflow cannula alignment and the risk of intraventricular thrombosis

**DOI:** 10.1186/s12938-021-00884-6

**Published:** 2021-05-11

**Authors:** Michael Neidlin, Sam Liao, Zhiyong Li, Benjamin Simpson, David M. Kaye, Ulrich Steinseifer, Shaun Gregory

**Affiliations:** 1 Department of Cardiovascular Engineering, Institute of Applied Medical Engineering, Helmholtz Institute, RWTH Aachen University, Pauwelsstraße 20, 52074 Aachen, Germany; 2 Department of Mechanical and Aerospace Engineering, Monash University, Clayton, VIC 3800 Australia; 3 Cardio-Respiratory Engineering and Technology Laboratory (CREATElab), Baker Heart and Diabetes Institute, Melbourne, VIC 3004 Australia; 4 Institute of Health and Biomedical Innovation (IHBI), Queensland University of Technology (QUT), Kelvin Grove, QLD 4059 Australia; 5 Department of Engineering, Nottingham Trent University, Clifton Lane, Nottingham, NG11 8NS UK

**Keywords:** Heart failure, Cardiomyopathy, Cardiovascular surgery, Thrombosis, Computational fluid dynamics, Left ventricular assist device, Cannula position

## Abstract

**Background:**

Adverse neurological events associated with left ventricular assist devices (LVADs) have been suspected to be related to thrombosis. This study aimed to understand the risks of thrombosis with variations in the implanted device orientation. A severely dilated pulsatile patient-specific left ventricle, modelled with computational fluid dynamics, was utilised to identify the risk of thrombosis for five cannulation angles. With respect to the inflow cannula axis directed towards the mitral valve, the other angles were 25° and 20° towards the septum and 20° and 30° towards the free wall.

**Results:**

Inflow cannula angulation towards the free wall resulted in longer blood residence time within the ventricle, slower ventricular washout and reduced pulsatility indices along the septal wall. Based on the model, the ideal inflow cannula alignment to reduce the risk of thrombosis was angulation towards the mitral valve and up to parallel to the septum, avoiding the premature clearance of incoming blood.

**Conclusions:**

This study indicates the potential effects of inflow cannulation angles and may guide optimised implantation configurations; however, the ideal approach will be influenced by other patient factors and is suspected to change over the course of support.

**Supplementary Information:**

The online version contains supplementary material available at 10.1186/s12938-021-00884-6.

## Background

Despite favourable outcomes and technological advances of left ventricular assist devices (LVADs), many complications still exist [1]. The source of thromboemboli responsible for ischaemic strokes may originate from a number of sites [2–4]. Non-physiological intraventricular flow patterns generated by the LVAD inflow cannula can be a contributing factor to thrombosis [1, 5–8]. However, a common clinical goal for LVAD cannula placement is the prevention of occlusion and is not typically oriented to minimise flow-induced intraventricular thrombosis [9–11]. Numerical studies have suggested that thrombosis can be affected by a range of variables including, surgical technique to patient-specific anatomy and pump orientation [12, 13]. Clinical reports have highlighted the significance of the LVAD inflow cannula angle. For instance, the risk of HeartMate II pump thrombosis increases, and the rate of survival decreases if the angle between the inflow cannula and centre of the LVAD rotor is acute (< 55°) [10, 14]. In particular, thrombosis was observed when the inflow cannula of a HeartMate II was directed towards the septum, which was attributed to increased suction events (leftward septal deviation leading to direct suction of the septum into the inflow cannula) and unfavourable intraventricular flows [15].

There have been limited studies assessing inflow cannula positioning; however, numerical studies have been undertaken [16]. A generic inflow cannula was apically implanted at angles of − 14°, − 7°, 0°, + 7°, + 14° relative to the left ventricular long axis. Utilising a static left ventricle with a non-existent mitral valve, a Lagrangian approach (injection of discrete particles) was implemented to assess the risk of thrombosis. It was reported that cannulation angles between ± 7° reduced the risk of thrombosis, based on reduced accumulated shear stress and residence time.

Clinical evaluation of LVAD inflow cannulation angles has typically been performed retrospectively, often as a result of unintended pump migration or mispositioning: conclusions are formed based on symptomatic clinical observations in contrast to understanding the problem. To better understand the relationship between LVAD inflow cannulation angle and thrombosis potential, this study addresses some limitations of previous numerical models such as neglecting the mitral valve, use of a rigid heart wall and the use of particles to evaluate the risk of thrombosis.

The presented study aimed to evaluate the risk of thrombosis in a pulsatile patient-specific left ventricle with variations in inflow cannula angles. Based on prior understandings of intraventricular flow under LVAD support [6, 7], it was hypothesised that a cannulation angle that promoted natural intraventricular flow patterns would be beneficial to reduce the risk of thrombosis. Five metrics were implemented to assess the risk of thrombosis: blood residence time, left ventricular washout, kinetic energy, instantaneous blood stagnation and pulsatility indices [3, 17, 18].

## Results

### Intraventricular flow dynamics under LVAD support

**Fig. 1:**
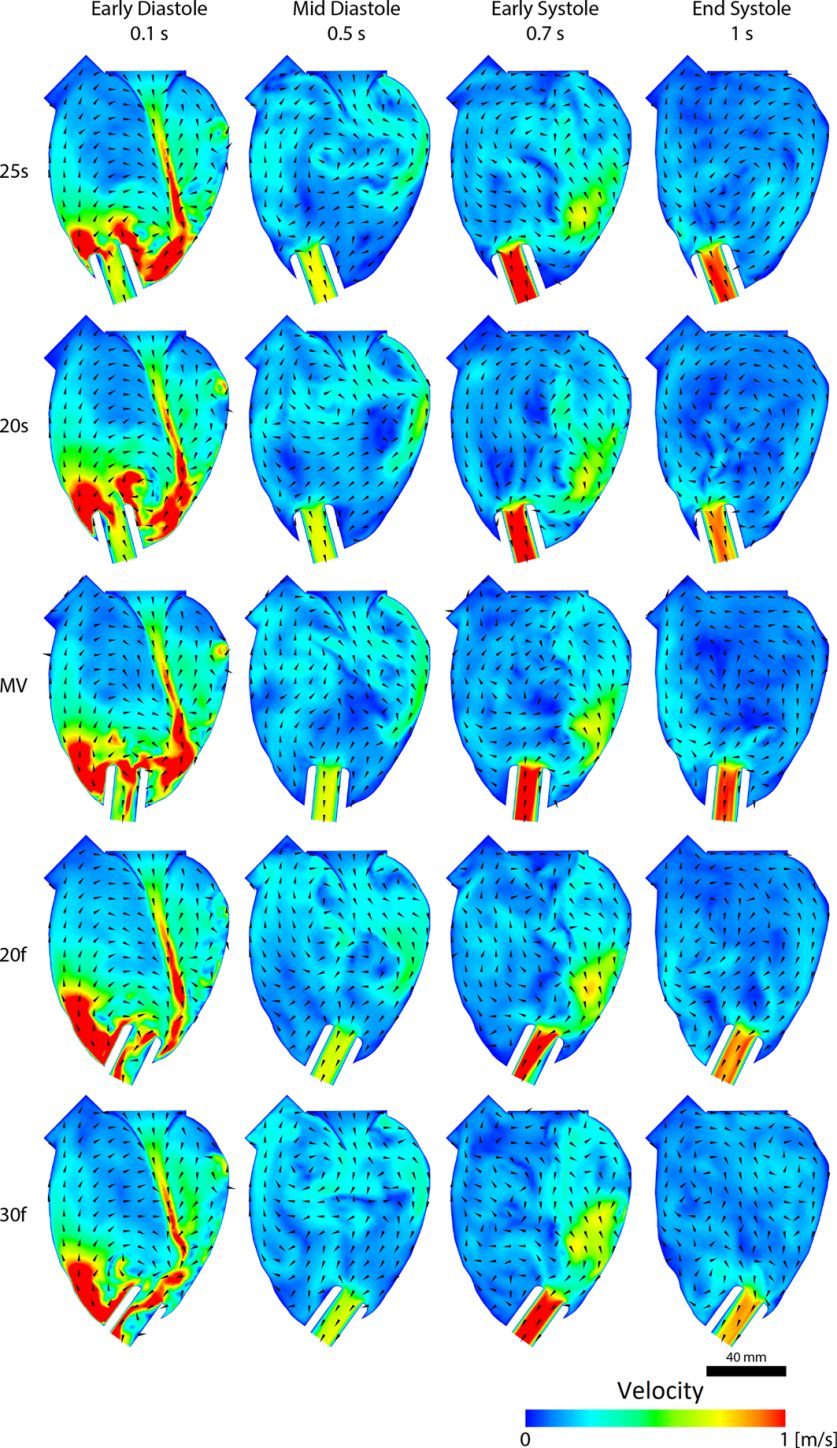
Intraventricular velocity contours. Severe left ventricular assist device inflow cannula angulation towards the septum (top row) and the free wall (bottom row) over a cardiac cycle. Refer to Fig. 1 for the corresponding label to the studied scenario

Intraventricular velocity contours for early diastole and mid-diastole as well as early and end-systole are shown in Fig. 1. Cannulation towards the septum was at 25° and 20°, assigned as 25 s and 20 s, respectively. Angulation towards the free/lateral wall at 20° and 30° were assigned as 20f and 30f. The 20° angulation to either side was chosen to emphasise the effects of inflow cannula alignments. All cannulation angles resulted in a diastolic vortex despite severe angulation towards the free wall. Inflow cannula angulation towards the free wall appeared to guide the incoming flow towards the inflow cannula tip, as shown in early diastole and systole in Fig. 1. At end-systole, cannulation angles towards the septum had greater uniformity of velocity vectors that contributed to a larger clockwise vortex, whereas cannulation angles towards the free wall showed multiple small clockwise vortices.

### Blood residence times

**Fig. 2:**
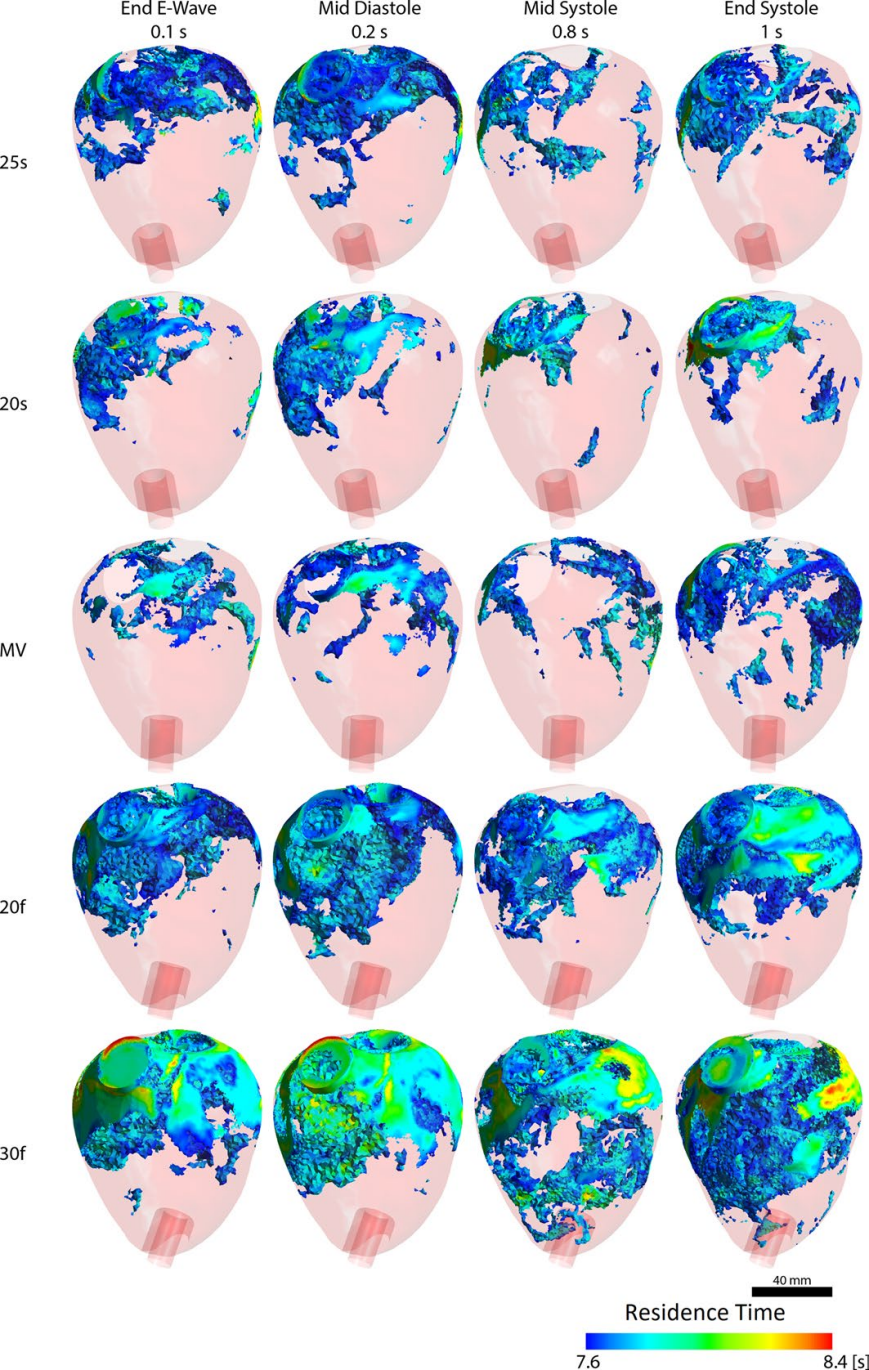
3D identification of long residence time regions with different cannulation angles. Long blood residence times are not limited to the left ventricular outflow tract, especially around the anterior and posterior aspects of the basal region. The shaded pink areas are for visualisation of the left ventricle

Average blood residence times in the left ventricle were higher when the inflow cannula was angled towards the free wall, as shown in Table 1. In reference to the MV case (MV); 25 s, 20 s, 20f and 30f had a difference of 0.36%, − 0.58%, 2.43% and 4.68%, respectively. While minor angling the inflow cannula 20° towards the septum was found to have the lowest blood residence time while further angulation to 25° increased blood residence times. The basal regions, the regions around the mitral and aortic valves, had the longest blood residence times. Long blood residence times did not only occur around the left ventricular outflow tract, but also at the anterior and posterior aspects of the basal region, as shown in Fig. 2.

**Table 1:** Quantitative metrics to predict the risk of thrombosis

Analysis metric	Models				
	25 s	20 s	MV	20f	30f
Blood residence time [s]	6.66	6.60	6.64	6.80	6.95
Ventricular washout after four cardiac cycles [%]	56.81	56.78	57.81	58.09	58.29
Peak kinetic energy density of left ventricle [J/m^3^]	326.04	325.74	328.42	325.91	324.67

### Ventricular washout and kinetic energy

**Fig. 3:**
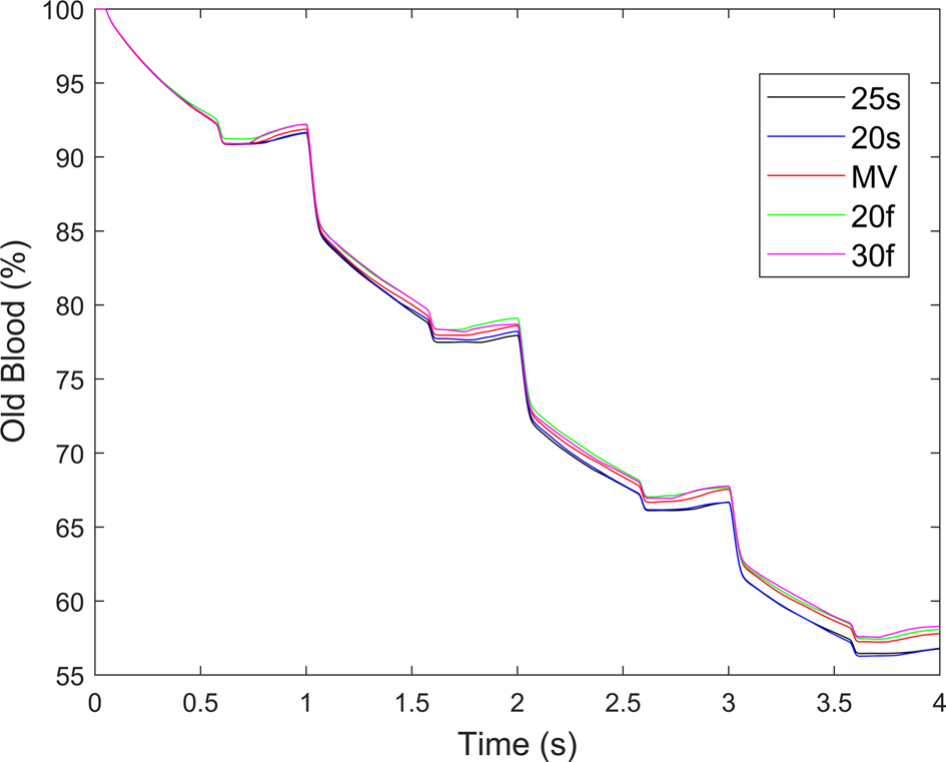
Rate of ventricular washout with different cannula angles. The rate of washout increased with angulation towards the septum. Ventricular washout describes the rate that old blood within the left ventricle is replaced with incoming blood from the left atrium

**Fig. 4:**
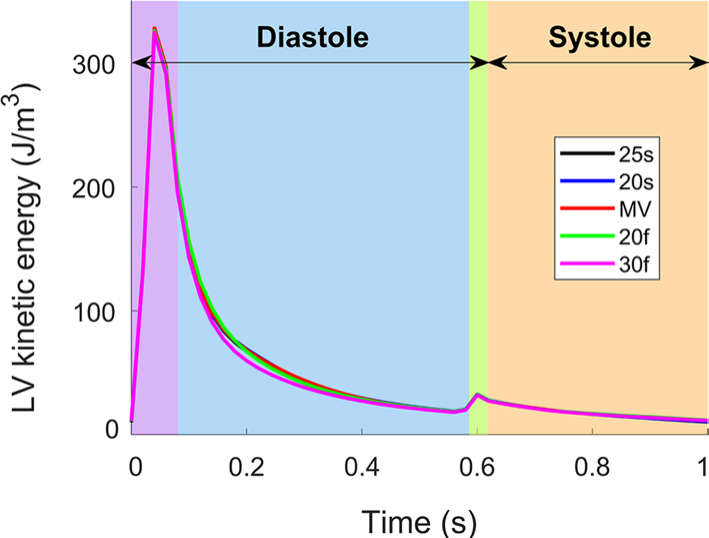
Mean left ventricular kinetic energy density of four cardiac cycles. The purple, blue, green and orange shaded areas correspond to the E-wave, mid-diastole, A-wave and systole, respectively

The rate of ventricular washout after four cardiac cycles increased when the inflow cannula was angled towards the septum, as seen in Fig. 3 and Table 1. The largest difference after four cardiac cycles was 1.51%. The variations in the alignment of incoming blood into the inflow cannula can also be seen in the Additional file 1: Fig. S1. The kinetic energy densities throughout the cardiac cycle are shown in Fig. 4 and the peak values are shown in Table 1. It can be seen that different cannula angulation does not influence either the peak kinetic energy density or the cycle-dependent energy density, with only negligible differences in the diastole.

### Pulsatility index

**Fig. 5:**
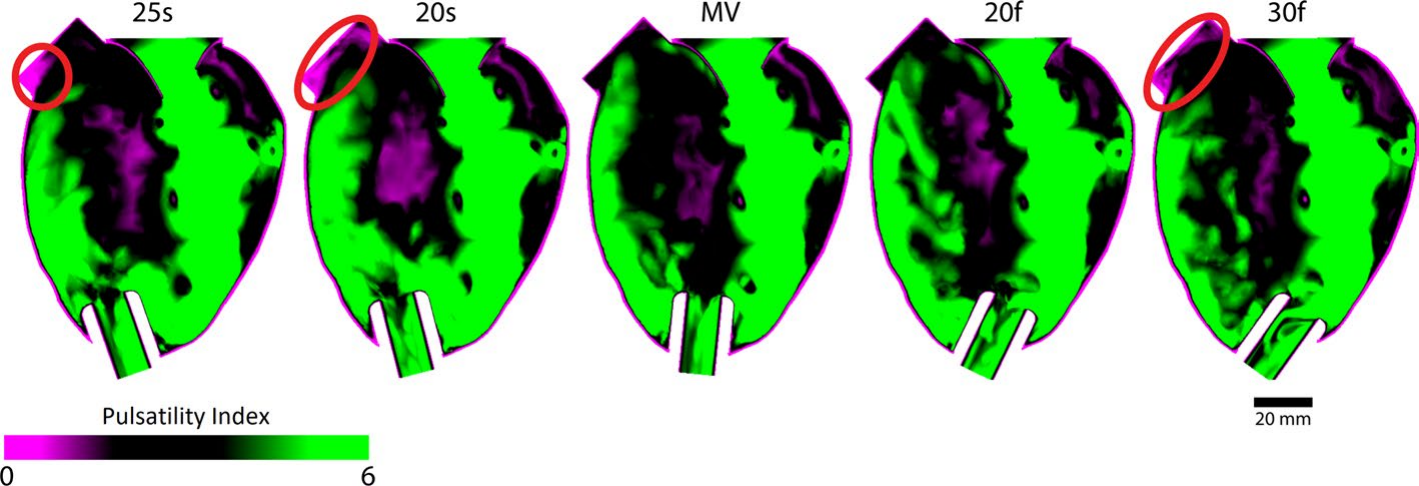
Pulsatility index map with different cannulation angles derived from four cardiac cycles. Inflow cannula alignment towards the septal or free wall resulted in low pulsatility around the left ventricular outflow tract as indicated by red circles

The pulsatility index contour plot (Fig. 5) indicated that if the cannula was aligned towards the septum, a low pulsatility index (PI) was found at the left ventricular outflow tract (PI < 1). Similarly, if the angulation was in close apposition to the free wall, then a low pulsatility index also occurs at the left ventricular outflow tract. Cannula alignment towards the mitral valve and free wall both had greater pulsatility indices at the left ventricular outflow tract (PI 2–3). The relative thickness of high pulsatility indices along the septal wall appeared to decrease as cannulation alignment rotated towards the free wall.

## Discussion

The risk of intraventricular thrombosis due to changes in inflow cannula cannulation angles implanted at the left ventricular apex was investigated. Based on the modelled scenario, this study determined whether the common cannula alignment towards the mitral valve is best practice and evaluated potential consequences when deviations occurred. We used a numerical approach going beyond the existing computational models considering ventricular wall movement, a mitral valve geometry and the surrounding cardiovascular system by means of a lumped parameter network. Results based on the blood residence time, rate of left ventricular washout, kinetic energy and pulsatility indices elucidated that inflow cannula alignment towards the mitral valve resulted in potentially more favourable flow dynamics than severe angulation towards either the septum or free wall.

From clinical observations of acute angles between the HeartMate II pump body and inflow cannula, it was difficult to determine the exact position of the inflow cannula relative to internal left ventricular features from the presented literature [10, 14, 19]. Nevertheless, this study assumed that an acute inflow cannula to pump angle might have the highest probability of being directed towards the septum. The increased risk of pump thrombosis due to this angulation could be correlated with the 25 s case, whereby the total blood residence time was 0.8% higher than inflow cannula angulation towards the mitral valve. However, angulation towards the free wall was found to be worse than septal alignment as blood residence times were up to 5% longer than mitral valve alignment. Considering the small differences, the clinical observation of increased pump thrombosis may not be solely attributed to the inflow cannula angle regarding the total blood residence time; however, it may have a cumulative effect with other factors. These include the biochemical elements of thrombus formation such as platelet activation by tissue factor and the role of the coagulation cascade [20]. Further aspects are the cannula design and insertion length as well as clinically relevant parameters like excessive LVAD speed, LV size and lack of aortic valve opening [16].

Longer blood residence times were consistently observed around the left ventricular outflow tract, but with cannula angulation towards the free wall, the LV anterior and posterior basal regions had increased volumes of prolonged residence times, highlighting the importance of assessing the entire left ventricular volume and not only in a single plane or section [8, 21]. Despite arguably small differences in blood residence times (~ 5%) and the rate of washout (~ 1%), it provided an interesting perception of the effects of cannulation angles. Even the slightest improvement to intraventricular flow could be advantageous considering that small thrombi may be conducive for the formation of larger structures due to a cumulative effect [3, 22].

Orientating the inflow cannula towards the free wall may increase the probability of inducing shorter flow paths between the left atrium and inflow cannula tip. The idea to increase the flow path between the mitral valve and inflow cannula tip, to preserve the natural clockwise vortex, has the potential to reduce the risk of thrombosis [6, 7]. Even though alignment towards the septum was more favourable than the free wall, if the inflow cannula long axis intersected the septum, then lower pulsatility indices were observed at the left ventricular outflow tract along with higher blood residence times. Reduced pulsatility indices for when the inflow cannula is angled towards the septum has been previously reported in a PIV study [23]. Based on the results, it is recommended to avoid alignment of the inflow cannula towards the septum, which would also avoid inflow cannula occlusion. The maximum angulation limit towards the septum could potentially be parallel to the septal wall, which may also prevent septal shift at higher pump speeds and reduce the likeliness of left ventricular suction, similar to suggestions by Chivukula et al. despite modelling differences [16].

Chivukula et al. indicated that when the inflow cannula was angled closer to alignment with the LV long axis, flow from the mitral valve to the inflow cannula was more direct [16]. However, this may not be typical for patients with dilated cardiomyopathy: the mitral jet is often directed more towards the free wall [24]. Furthermore, it was speculated that the direct flow between the mitral valve and inflow cannula inlet was favourable, contrary to the recommendations presented here. Due to the evaluation method of injected particles using a Lagrangian approach, the direct flow from the mitral valve to the inflow cannula does not entirely explain the risk of intraventricular thrombosis. Perhaps due to the smaller simulated left ventricular geometry, the effects may not be as pronounced in capturing recirculation zones, hence similar conclusions.

Attenuation of vortex structures due to cannulation orientation has similarly been reported [5]. The previous in vitro study suggested that when an inflow cannula was aligned towards the left ventricular outflow tract, more distinct vortices were formed, corroborating with the presented results in our study. Comparing the velocity vectors between 25 s and 30f in Fig. 1 at end-systole, a large uniform clockwise fluid rotation was present when the inflow cannula was angled towards the septum and mitral valve. In contrast, when the inflow cannula was angled towards the free wall, multiple smaller vortices were present.

The pulsatility index contour plot (Fig. 5) indicated that 25 s, 20 s and 30f had low indices around the left ventricular outflow tract, which can be related to the blood residence time visualisation in Fig. 2. Interestingly, the pulsatility index contour plot of the mitral jet can also be associated with the rate of ventricular washout (Fig. 3): the high pulsatility regions due to the mitral jet can be seen to align with the inflow cannula, especially with angulation towards the free wall (20f and 30f); therefore, potentially explaining the fast but detrimental clearance of incoming blood. The calculated pulsatility indices correspond with the results obtained in the in vitro study [3] with values between 0 and 6 throughout the left ventricle, values below 1 at the left ventricular outflow tract and values > 5 in most parts of the left ventricle. It has to be noted, that pulsatility index is a spatially resolved value and, if the interest lies in obtaining a spatially averaged parameter, the blood residence time should be used instead.

The most significant finding in this study was in agreement with Laumen et al. [5]. There, it was suggested that the ideal inflow cannula placement would be parallel to the septal wall. It was thought that an inflow cannula position pointed towards the septal wall would result in a large uniform vortex, which was found in the presented study. Nevertheless, the alignment of inflow cannulae is not solely related to cannula angulation at the apex, but also the site of cannulation and patient-specific left ventricular geometry.

The results elucidated the importance of cannulation angles, emphasising the significance to minimise early clearance of incoming blood during diastole. However, consideration of endocardial contact and suction should not be overlooked. It is expected that no ideal inflow cannula angle, length or site exists for all patients, but instead will be dependent on the patient condition, such as the degree of left ventricular dilation.

### Limitations

Limitations of the model have been previously described, which included the assumption of smooth endocardial walls; ventricular movement in a balloon-like manner based on reduced twist [25]; and neglected internal left ventricular features [26]. Laminar flow was assumed, however the flow might be in the transitional/turbulent regimen, necessitating turbulence modelling. The effect of this simplification on the thrombus risk metrics should be evaluated. The assumed heart rate of 60 bpm is low for VAD patients and may not represent typical patients and only one patient-specific geometry was investigated. Thus, it must be remembered that this paper only presents one potential scenario and the results must be interpreted with caution. All the evaluated metrics indirectly predict the risk of thrombosis and are only an indicator of blood stagnation. A quantifiable relation of these haemodynamic parameters (blood residence time, washout, pulsatility index) towards thrombus formation if difficult to determine as spatially resolved experimental data on thrombus formation in the LV during LVAD support is scarce to non-existent. Each metric has its advantages and disadvantages. Blood residence time, ventricular washout and kinetic energy are spatially averaged metrics allowing for a quick comparison, however neglecting differences within the LV. On the other hand, pulsatility index and the ventricular flow field can be resolved spatially (and if required temporally), however a quantitative comparison for several cases might become difficult. Thus, rather a combination of these metrics should be considered when interpreting the results and we do not believe that there is one single value that can serve as a surrogate marker for flow-induced thrombus formation.

With the awareness of limitations intrinsic to numerical studies, the direct clinical significance is difficult to predict. However, further insights provided into intraventricular flow characteristics due to changes in cannulation angles may provide additional aspects of consideration when designing and implanting LVADs. Current clinical principles for inflow cannula alignment is to angle the inflow cannula parallel to the septum and towards the left ventricular centre, which is in agreement with the suggestions presented in this study [27]. However, the presented findings may be limited to the simulated patient condition; therefore, other haemodynamic and spatial combinations should be pursued. Since no aortic valve opening was simulated and atypical to clinical preference, it is hypothesised that superior ventricular washout could be obtained in the basal region if aortic valve opening is promoted; however, parallel septal alignment is expected to be superior based on the avoidance of early blood clearance and higher kinetic energies at the LV apex.

In order to leverage clinical significance, future applications of this numerical model should focus on a higher number of patient-specific geometries. One case study, as shown within this work, is not able to quantify the role of flow alterations and provide statistically significant results with a clinical implication. Investigations of many geometries require much higher computational costs, a problem that needs to be tackled as the next step. We believe that reduction of model complexity under simultaneous considerations of the thrombus risk metrics presented herein, should be performed. In other words, one should gradually simplify the model and compare the changes in the outcomes such as pulsatility index, blood residence time and intraventricular vortices. Possible simplifications could be steady-state simulations at some time points instead simulations of the full cardiac cycle, a rigid wall assumption instead of a fluid–structure interaction simulation or the disregard of the lumped parameter network model.

The results from our study can be used as a reference case to see, if model simplifications violate the prediction of numerical models focussing on thrombus risk in the left ventricle during LVAD support.

## Conclusions

Understanding the effect of inflow cannulation angle on intraventricular flow is non-trivial in vivo; thus, this study endeavoured to provide insights into the effect of inflow cannulation angles on intraventricular flow numerically. Notwithstanding the model limitations, the ideal inflow cannula alignment, likely to be patient and device-dependent, should be positioned in a manner to prevent occlusion and the premature clearance of blood directly entering the left ventricle. The ideal inflow cannula alignment in this simulated patient condition was angled towards the mitral valve and up to parallel to the septum.

## Methods

The numerical simulation (Fluent, ANSYS 18.0, PA, USA) consisted of three coupled components: (1) a zero-dimensional lumped parameter network providing the boundary conditions to the computational fluid dynamics simulation; (2) a fictitious heart wall with a pressure loading to provide heart wall motion; and (3) a severely dilated patient-specific left ventricle for the three-dimensional computational fluid dynamics model. The coupling of these components have been extensively described elsewhere [6, 7]; however, a summary is provided here.

### Lumped parameter network model

**Fig. 6:**
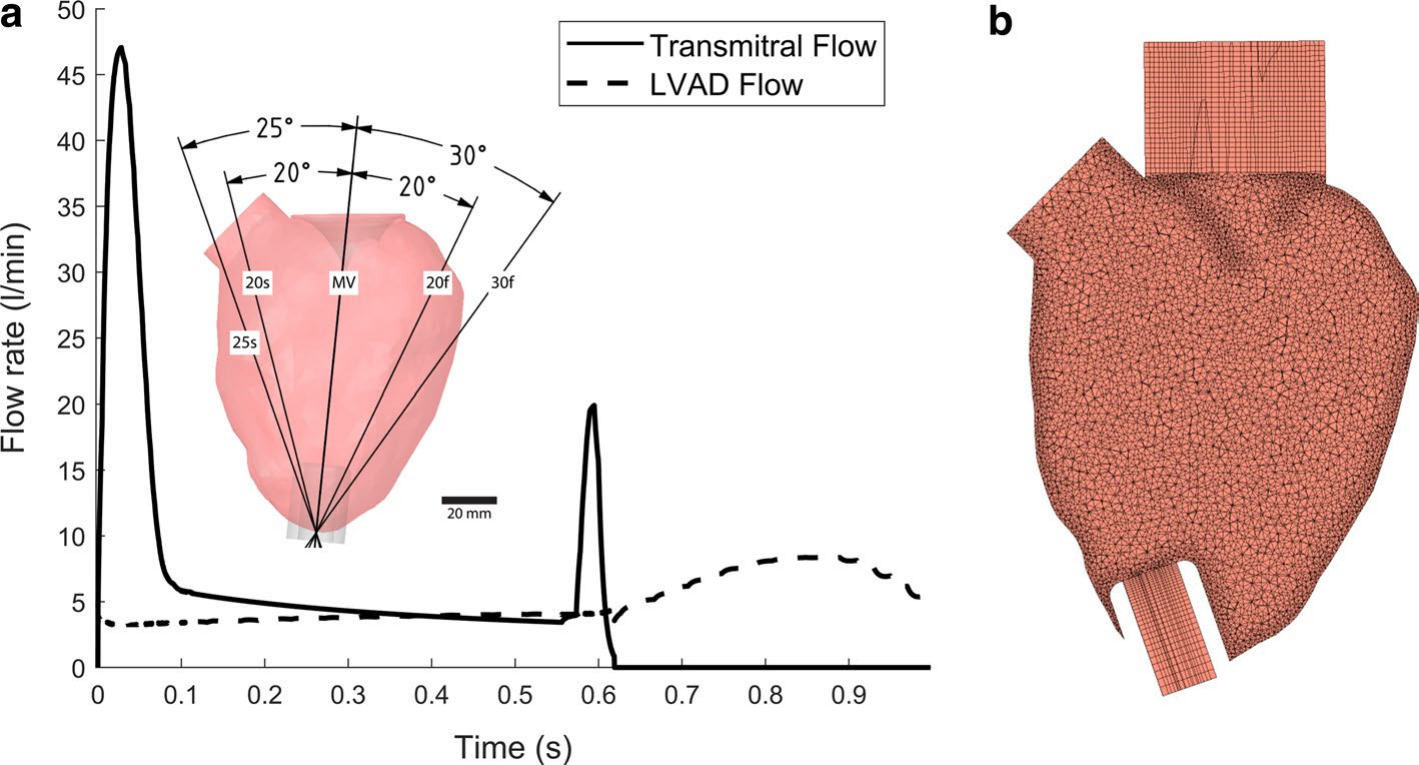
Evaluated inflow cannula angles, boundary conditions and mesh. **a** The alignment of the inflow cannula towards the mitral valve was used as the reference case. The flow rates were driven by a lumped parameter network (LPN) that was one-way coupled to the computational fluid dynamics simulation. **b** Computational mesh for the 25 s scenario

The cardiovascular system was represented in a zero-dimensional form by a series of ordinary differential and algebraic equations that were represented with ten compartments [6, 28]. Each vessel compartment was described using a two-element (resistance and compliance) Windkessel model. The model simulated a heart failure condition under LVAD support by targeting a cardiac output of 5 L/min and a mean aortic pressure between 75 and 90 mmHg. The LVAD was based on a HeartWare HVAD running at a constant speed of 2600 rpm. The transmitral flow calculated from the lumped parameter network model was one-way coupled to the 3D computational fluid dynamics model at the mitral valve inlet. A more detailed description of the lumped parameter network model together with the chosen parameters is included in the Additional file 1. Figure 6a shows the transmitral flow and the LVAD flow as computed by the lumped parameter network model.

### Heart wall motion

The heart wall motion was simulated by loading a fictitious left ventricular wall with a time-dependent pressure profile, which was based on the left ventricular stroke volume simulated from the lumped parameter network model, as previously presented [7]. The deformation of the wall was transferred to the mesh of the fluid domain in a one-way coupled manner.

### Left ventricular geometry

The left ventricular geometry, reconstructed from computed tomography, was from an LVAD candidate with a severely dilated LV; see [7]. A parametric mitral valve was placed in the anatomical position with guidance from the medical imaging data. The mitral valve state was coupled to the lumped parameter network model which triggered the valve to be either on or off. Five different cannulation angles with an insertion length of 24 mm were evaluated (Fig. 6). The reference angle was defined to be when the inflow cannula was in alignment with the mitral valve, assigned as MV, as typically seen in the clinic [29]. Two angles to the septal (25° and 20°–25 s, 20 s) and lateral sides (30° and 20°–30f, 20f) angled on the coronal plane were studied. The difference in maximum angulation, 25 s vs. 30f, representing severe mispositioning between the two sides was restricted by the inflow cannula endocardial contact. For the simulated heart failure condition, the aortic valve did not open to simulate the worst-case scenario.

### Computational fluid dynamics model

Meshing strategy and element sizes were chosen on previous mesh convergence studies (blood residence time and rate of washout were taken as metrics for mesh independence) [7]. The model had approximately one million cells with a max. skewness of 0.9 and min. mesh orthogonal quality of 0.28. The ventricle was meshed with unstructured tetrahedral elements with a size of 2e−3 m. The mesh was refined at the ventricular walls, the mitral valve and the cannula by enabling mesh defeaturing with a size of 5e−4 m. The inlet domain of the model and the inlet cannula were meshed with hexahedral elements with a size of 2e−3 m through sweeping operations. Figure 6b shows the mesh for the 25 s case. The models were initialised for 30 s, and then 4 s were simulated for the analysis of the results at a heart rate of 60 beats per minute. The density of blood was assumed to be 1060 kg/m^3^. A Carreau model was used to model the non-Newtonian behaviour of blood [30]. Laminar flow was assumed, as the average Reynolds number was below 1000 as measured at the mitral inlet. Existence of turbulence has been a debate and other CFD studies have also assumed laminar flow [31, 32]. The PISO algorithm was used to solve the Navier–Stokes equations. Temporal discretization was performed with a second-order implicit scheme. Spatial discretization for the momentum was second-order upwind and for the pressure the pressure staggering option was selected. The deformation of the fluid mesh due to the heart wall motion was treated with diffusion-based smoothing to prevent the collapse of the mesh elements. The scaled convergence criteria were set to 10–5 for *x*/*y*/*z* velocity and continuity. The total CPU time was 6240 core-hours per model, excluding initialisation, with an Intel Xeon 2.5 GHz processor.

### Analysis of thrombus risk

Currently, there is no gold-standard for the numerical prediction of thrombosis; however, various metrics have been previously utilised to predict the risk of thrombosis based on the concept of blood stasis. Therefore, four metrics have been implemented to assess the risk of thrombosis, including blood residence time, ventricular washout, kinetic energy and the pulsatility index [3, 17–19]. Our previous studies [6, 7] explain the implementation in more detail.

Regions of relatively stagnant blood can be evaluated by the time that blood resides within the left ventricle. The blood residence time was evaluated with an Eulerian approach, whereby all domains were initialised to 0 s. Even though no diffusion was assumed, the Eulerian approach intrinsically has numerical diffusion. The simulations were initialised until the blood residence time reached a steady state with each cardiac cycle. The mean blood residence time in the left ventricular volume was time-averaged over four cycles. The blood residence time analysis provides a global insight into the blood residence time dynamics.

Closely related to blood residence time, ventricular washout is the rate of replacing old blood with “fresh” incoming blood from the left atrium into the left ventricle [17]. The difference between blood residence time and ventricular washout is that ventricular washout provides insights into the time-dependent flow paths. A fast rate of ventricular washout could reduce the risk of thrombosis by reducing blood stasis. Ventricular washout was simulated using a virtual ink concept. All fluid domains were initialised with an ink concentration of 0 and the incoming ink 1, representing fresh blood entering from the left atrium. The identification of incoming blood was activated after flow initialisation. The percentage of old blood in the ventricle overtime was normalised by the left ventricular volume.

High kinetic energy within the left ventricle may be beneficial to reduce the risk of thrombosis due to increased blood motion and kinetic energy can be connected to the functional performance of the ventricle [33]. The cycle-dependent kinetic energy was calculated from the volume-averaged dynamic pressure and averaged over four cycles.

The pulsatility index indicates the maximum flow velocity variations in a localised area, where high pulsatility indices may signify greater acceleration/deceleration of blood, thus a lower probability of blood stasis [3]. The pulsatility index evaluated the difference between the maximum and minimum velocity of each pixel in the plane of interest over four cardiac cycles at a sample rate of 0.02 s. The difference was normalised by the volume-average mean velocity over the cardiac cycles and was calculated according to Eq. (1):

1 $$\mathrm{PI}=\frac{{v}_{\mathrm{max}}-{v}_{\mathrm{min}}}{{v}_{\mathrm{ave}}}.$$

## Supplementary Information

**Additional file 1**: Includes supplementary methods and results.

## Data Availability

Available upon request.
