# Supplementary material for: Understanding the influence of left ventricular assist device inflow cannula alignment and the risk of intraventricular thrombosis
Source: Biomed Eng Online. 2021 May 11;20:47. doi: 10.1186/s12938-021-00884-6 (PMC8114696; doi:10.1186/s12938-021-00884-6)
Supplement: Supplementary file 1 — Additional file 1: Includes supplementary methods and results. [file 12938_2021_884_MOESM1_ESM.docx]

**Additional File 1**

*Supplementary methods*

**Lumped parameter model (LPM)**

The LPM consists of 13 compartments. Ten compartments describe the cardiovascular circulation – left and right atrium, left and right ventricle, aorta, system arterial and systemic venous circulation, pulmonary artery, pulmonary arterial and pulmonary venous circulation. In addition, the VAD is described by an inflow cannula, an outflow cannula and the pump compartment which creates a relationship between pressure head and pump flow.

Except for the cardiac chambers and the pump, all remaining compartments (8 compartments) were described with a resistance R and a hydraulic compliance C in a parallel circuit yielding the following ODEs (equation 1a and 1b):

$Q_{out}=\frac{p_{i}-p_{i+1}}{R_{i}}$ (1a)

$\frac{{dp}_{i}}{dt}=\frac{Q_{in}-Q_{out}}{C_{i}}$ (1b)

Q_in_ and Q_out_ are the incoming and outgoing flows into the compartment *i* (R_i_ and C_i_); p_i_ and p_i+1_ are the pressures of the corresponding and the downstream compartment, respectively.

The ventricles (equation 2a) and atria (equation 2b) were modelled as time-varying elastances described by the following equations:

$p_{j}= V_{j}e_{v}\left( t \right)E_{max,j}+(1-e_{v}\left( t \right))(\alpha_{j}e^{\kappa_{j}V_{j}}+\beta_{j})$ with j= LV or RV (2a)

$p_{k}=V_{k}(e_{a}\left( t \right)\left( E_{max,k}-E_{d,k} \right)+E_{d,k})$ with k =LA or RA (2b)

$\frac{{dV}_{i}}{dt}=Q_{in}-Q_{out}$ (3)

e_v_(t) and e_a_(t) are the ventricular and atrial activity functions (analytical formula in equation 4). E_max_ and E_d_ are the maximal systolic and diastolic elastances. α, β and κ are parameters quantifying end-diastolic ventricular elastance.

T_s1,v/a_ and T_s2,v/a_ are the respective time constants of the ventricular and atrial activity functions. T_c_ is the duration of the cardiac cycle (1 s).

$e_{v/a}=\left\{ \begin{aligned} \frac{1-cos(\frac{t}{T_{s1,v/a}}\pi)}{2}, 0\leq t <T_{s1,v/a} \\ \frac{1+\cos\left( \frac{t-T_{s1,\frac{v}{a}}}{T_{s2,\frac{v}{a}}-T_{s1,\frac{v}{a}}} \right), T_{s1,v/a}\leq t<T_{s2,v/a}}{2} \\ 0, T_{s2,v/a}\leq t<T_{c} \end{aligned} \right.$ (4)

The valves (aortic, tricuspidal, mitral, pulmonary) were described as an ideal diode allowing only forward flow direction and an orifice with a quadratic pressure loss (loss coefficient CQ_i_), see equation 5:

$Q_{out}=\sqrt{p_{i}-p_{i+1}}{CQ}_{i}$ (5)

Considering the pressure difference H across the LVAD, the according blood flow through the pump is shown in equation 6. The parameters a_i_ were fitted to an existing HVAD HQ curve. rpm is the rotational frequency of the VAD:

$Q_{VAD}=a_{0}+a_{1}rpm+a_{2}H+a_{3}H rpm+a_{4}H^{2}$ (6)

The system of ODEs was implemented in MATLAB and solved with an explicit Euler algorithm for a duration of 10 seconds and a timestep of 1e-4 s. After confirmation of a steady state, the last cycle was taken as an input for the CFD model, see Figure 1 in main text.

The parameters of the LPM are shown below. They are mostly taken from the work of Neidlin et al. (reference 27 in the main text) and were adjusted to model the desired behavior.

Table 1: Model parameters

| **Part** | **Parameter** | **Value** |
| --- | --- | --- |
| *Systemic circulation* | R_aorta_ [mmHg s/ml]  C_aorta_ [ml/mmHg]  R_sart_ [mmHg s/ml]  C_sart_ [ml/mmHg]  R_svn_ [mmHg s/ml]  C_svn_ [ml/mmHg] | 0.015  0.17  0.845  1.1  0.04  12 |
| *Pulmonary circulation* | R_pa_ [mmHg s/ml]  C_pa_ [ml/mmHg]  R_part_ [mmHg s/ml]  C_part_ [ml/mmHg]  R_pvn_ [mmHg s/ml]  C_pvn_ [ml/mmHg] | 0.008  0.625  0.065  4.32  0.04  0.6 |
| *Right heart* | CQ_tri_ [ml/s mmHg^0.5^]  CQ_pul_ [ml/s mmHg^0.5^]  E_maxRA_ [mmHg/ml]  E_dRA_ [mmHg/ml]  E_maxRV_ [mmHg/ml]  α_RV_ [mmHg/ml]  β_RV_ [mmHg/ml]  κ_RV_ [1/ml] | 400  350  0.2025  0.1575  0.342  0.047  4.27  0.028 |
| *Left heart* | CQ_mi_ [ml/s mmHg^0.5^]  CQao [ml/s mmHg^0.5^]  E_maxLA_ [mmHg/ml]  E_dLA_ [mmHg/ml]  E_maxLV_ [mmHg/ml]  α_LV_ [mmHg/ml]  β_LV_ [mmHg/ml]  κ_LV_ [1/ml] | 400  350  0.45  0.2125  0.768  0.064  2.5  0.033 |
| *VAD* | a_0_  a_1_  a_2_  a_3_  a_4_  rpm | 28.1673  0.0738  -3.1436  6.75e-4  -0.0037  2600 |
| *Additional parameters* | T_c_ [s]  T_s1,v_ [s]  T_s2,v_ [s]  T_s1,a_ [s]  T_s2,a_ [s] | 1  0.3  0.45  0.04  0.09 |

*Supplementary results*

*
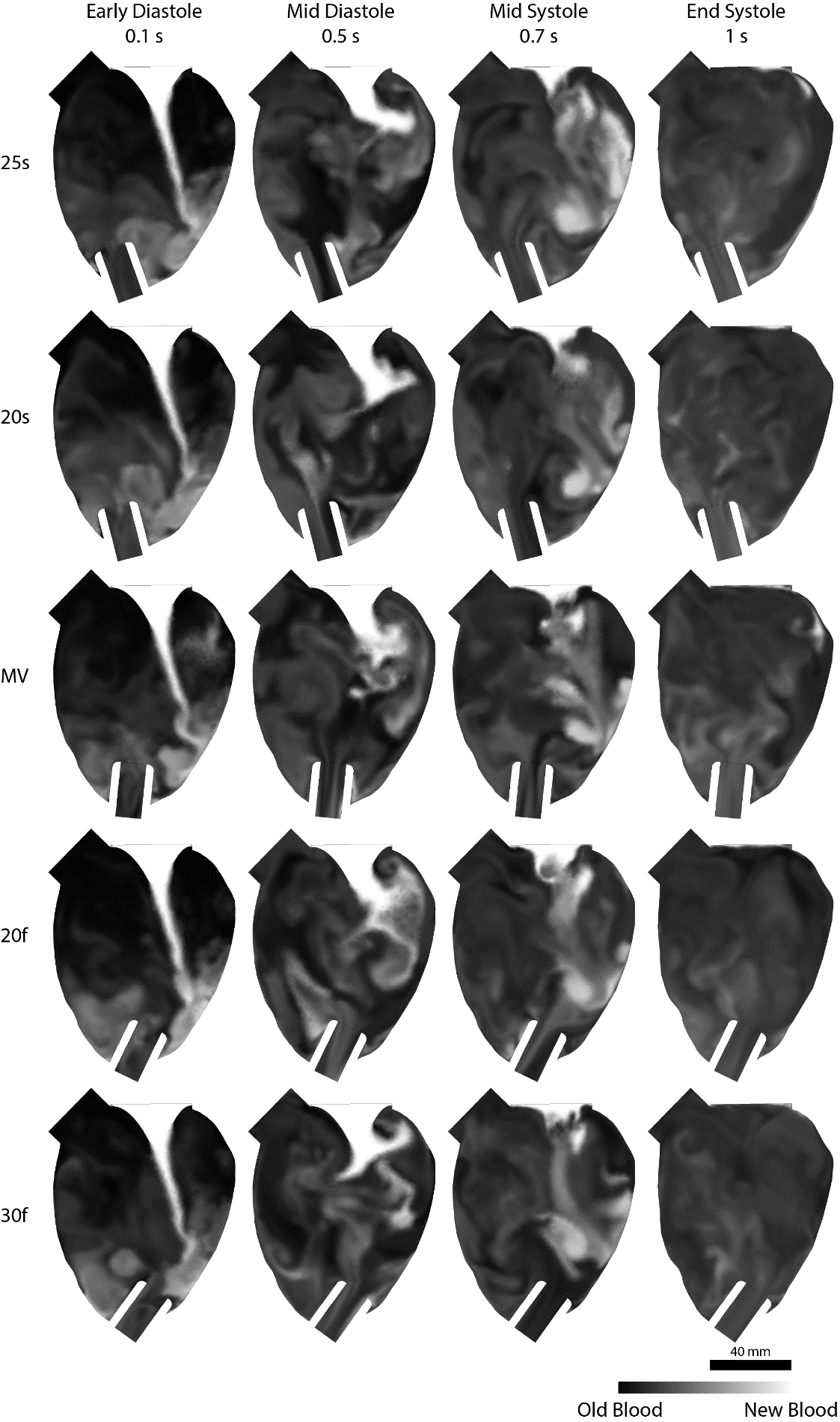
*

**Fig. SI1** Flow path visualisation of new incoming blood to replace old blood with two severe cannulation angles towards the septum and free wall. Cannula angulation towards the free wall had better flow alignment towards the incoming blood, potentially increasing the risk of thrombosis
